# A case of hepatic cyst-induced inferior vena cava thrombosis

**DOI:** 10.12669/pjms.303.4680

**Published:** 2014

**Authors:** Jaecheol Moon, Dahee Heo, Sanghoon Han

**Affiliations:** 1 Jaecheol Moon, Department of Internal Medicine, Jeju National University Hospital, Korea.; 2 Dahee Heo, Department of Internal Medicine, Jeju National University Hospital, Korea.; 3 Sanghoon Han, Department of Internal Medicine, Jeju National University Hospital, Korea.

**Keywords:** Anticoagulation, Hepatic cysts, Inferior vena cava, Thrombosis

## Abstract

A 92-year-old woman visited the hospital with edema of both lower extremities. Computed tomography revealed her inferior vena cava (IVC) was compressed by a massive hepatic cyst. A massive IVC thrombosis and pulmonary thromboembolism (PTE) were also observed. Medical treatment rather than radiologic intervention was preferred because of the patient’s advanced age and poor performance status. IVC thrombosis and PTE disappeared after 6 months of anticoagulation therapy. To the best of our knowledge, this is the first study in the English literature to report IVC thrombosis caused by congenital hepatic cysts that was treated without vascular intervention.

## INTRODUCTION

Congenital liver cysts are usually asymptomatic and do not require treatment. Most of these cysts are detected incidentally during radiologic imaging (ultrasonography or computed tomography) performed because of symptomatic events such as hemorrhage, rupture, obstructive jaundice, and thrombotic events including deep vein thrombosis.^1^^-^^3^

The Three most important factors that result in deep vein thrombosis are vein stasis, hypercoagulability, and endothelial injury.^4^ The physiological conditions that are risk factors for deep vein thrombosis are advanced age, bed-ridden status, and past history of thromboembolism, malignancy, surgical procedures, or trauma.^5^

Generally, deep vein thrombosis results in inferior vena cava (IVC) thrombosis. The occurrence of IVC thrombosis may be related to autosomal dominant polycystic kidney disease, pancreatitis, ovarian cysts, renal cell carcinoma, post-traumatic hematoma, or liver abscess.^6^^-^^8^ However, IVC thrombosis caused by solitary hepatic cysts is very rare. Moreover, once IVC thrombosis occurs, the clinical prognosis can be fatal. In most cases of hepatic cyst-induced IVC thrombosis, transluminal and interventional therapies such as filter insertion or venoplasty have been considered. However, none of the studies in the English literature in PubMed have reported on the treatment without vascular intervention for this thrombosis.

We present a case report of a patient with IVC thrombosis and pulmonary thromboembolism caused by congenital hepatic cysts, in whom anticoagulation therapy without the use of a vascular intervention such as an IVC filter was effective.

## CASE REPORT

**Fig.1:**
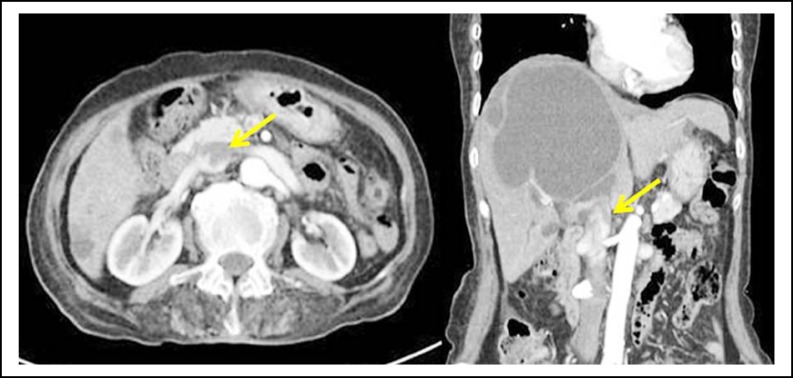
Computed tomography scan revealing multiple variable-sized cysts in both the kidneys and liver. The hepatic level of the IVC is compressed by massive liver cysts. IVC thrombosis is observed

A 92-year-old woman was admitted to our center because of severe edema of both lower extremities, loss of appetite, and general fatigue. Upon admission, her blood pressure was 128/78 mmHg, pulse rate was 69 beats/min, respiratory rate was 20/min, and her body temperature was 36C. Her laboratory data revealed no leukocytosis (white blood cell count 7800/µL). However, she had mild anemia (hemoglobin level, 11.1 g/dL) and increased levels of serum liver transaminase (alanine transaminase, 119 IU/L), alkaline phosphatase (943 U/L), and total bilirubin (8.6 mg/dL). The serum CA 19-9 concentration was elevated (61.15 U/mL). Abdominal computed tomography scan (CT) revealed multiple variable-sized cysts in both the kidneys and liver. The hepatic level of the IVC was compressed by massive liver cysts, and thrombosis of the infrarenal IVC and left iliac artery were observed (Fig.1). Abdominal CT revealed pulmonary artery thromboembolism.

Further tests were performed to check for hypercoagulability. Her prothrombin time and activated partial thromboplastin time were within the normal range. The patient fibrinogen degeneration product level was increased to 20 µg/mL, and her antithrombin III activity was normal. Protein C, S, and factor V Leiden tests revealed no abnormal findings. Anti-nuclear antibody and Lupus anticoagulant test result were negative. The patient’s serum was positive for anti-cardiolipin IgG (44.9 GPL units/mL) and anticardiolipin IgM (20.2 MPL units/mL). These results indicated a possibility of antiphospholipid antibody syndrome (APS). To confirm this disease, the tests need to be repeated after 3 months, but the patient refused further work-up. We considered aspiration or sclerotherapy for the massive hepatic cysts, but the patient refused invasive interventional therapy because of advanced age. Therefore, only anticoagulation therapy with enoxaparin was maintained.

**Fig.2:**
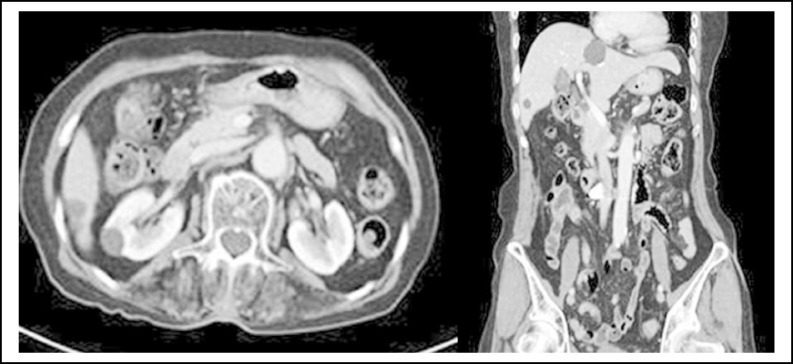
Eight weeks after anticoagulation therapy, computed tomography revealing decompression of the inferior vena cava and disappearance of inferior vena cava thrombosis

Two months later, a CT scan was performed for follow-up. Interestingly, on the CT scan, the huge hepatic cysts had decreased in size, although the reason for this reduction was not clear. CT also revealed IVC decompression but reduced hepatic cysts, along with the disappearance of the left iliac artery thrombosis and pulmonary thromboembolism. The therapeutic dose of enoxaparin (1 mg/kg every 12 h) was maintained for 6 months. In the status that hepatic cyst compressing IVC still existed, further maintenance of anticoagulation was suggested to the patient. However, the patient refused further enoxaparin injection because of discomfort and has maintained aspirin 100 mg for >24 months. Thus far, there is no sign of thrombosis recurrence.

## DISCUSSION

Congenital liver cysts are usually asymptomatic and incidentally discovered by imaging studies such as ultrasonography or CT. A single or multiple simple liver cysts can be found. These cysts can have sizes ranging from millimeters to 20 cm.^9^ Symptomatic cysts are rare but sometimes cause intracystic bleeding, rupture, or secondary bacterial infection. Compression of the IVC by cysts can result in edema in both extremities.

For treating patients with symptomatic hepatic cysts, aspiration, injection of a sclerosing agent into the cyst cavity, total cystectomy, hepatic resection, and orthotopic transplantation have been recommended.^10^^,^^11^ For IVC thrombosis treatment, surgical removal of blood clots, IVC filter insertion, and anticoagulation therapy are usually recommended.^12^^,^^13^ However, in our case, IVC thrombosis and pulmonary thromboembolism had completely disappeared with only the use of anticoagulation therapy and no vascular intervention.

Only 4 case reports in the English literature have presented IVC thrombosis caused by compression by simple hepatic cysts in patients without predisposing factors.^14^^-^^16^ In all 4 cases, patients required an interventional treatment in addition to anticoagulation therapy. Kashiwagi et al.^15^ reported a patient with massive hepatic cysts compressing the IVC and right ventricle, resulting in IVC thrombosis. Laparoscopic surgery was performed to aspirate the cyst. Leung et al.^16^ reported a patient with hepatic cysts with IVC compression and severe infection in whom cellulitis due to venous occlusion of the lower extremity progressed to systemic infection, and the patient later died. England et al.^17^ reported a patient in whom a massive hepatic cyst caused thrombosis in the IVC, right atrium, main pulmonary trunk, and left pulmonary artery, for which the patient underwent surgical removal of the thrombosis of the various sites.

In our case, although undiagnosed, the patient probably had APS as a predisposing factor for systemic thrombosis. However, even if this condition was diagnosed, the treatment for our patient would have been the same because IVC thrombosis caused only by APS is very rare,^18^ and because treatment for APS patients includes long-term anticoagulation.

We suggest close follow-up for patients with congenital liver cysts large enough to compress the IVC, and in cases of IVC thrombosis, conservative anticoagulation may be a feasible treatment option for elderly patients with poor performance status.
